# The combination of *Lactobacillus acidophilus* DSMZ 26280 and *Limosilactobacillus reuteri* DSMZ 25441 has an impact on clinical course and gut microbiota of children with acute infectious diarrhea

**DOI:** 10.3389/fmicb.2026.1792126

**Published:** 2026-06-08

**Authors:** Necibe Tugce Goktas, Sirin Guven, Ener Cagri Dinleyici

**Affiliations:** 1 Department of Pediatrics, Sancaktepe Ilhan Varank Research and Training Hospital, University of Health Sciences, Istanbul, Türkiye; 2 Department of Pediatrics, Eskisehir Osmangazi University Faculty of Medicine, Eskisehir, Türkiye

**Keywords:** acute infectious diarrhea, biotic, children, microbiota, probiotic

## Abstract

**Introduction:**

Previous studies and society guidelines have proposed probiotics as a complementary therapy for acute infectious diarrhea, which may shorten the disease course, yet strain-specific effects and microbiome correlates remain incompletely defined. We aim to evaluate the effect of a combination of *Lactobacillus acidophilus* and *Limosilactobacillus reuteri* on the duration of diarrhea and gut microbiota composition in children with acute infectious diarrhea.

**Patient and methods:**

In a prospective, randomized, controlled, open-label trial at a tertiary pediatric emergency department (March–August 2024), children aged 1–6 years with acute infectious diarrhea lasting less than 24 h were allocated 1:1 to standard therapy (oral rehydration ± intravenous fluids) with or without 5-day probiotic (*L. acidophilus* DSMZ 26280; 108 CFU) and (*L. reuteri* DSMZ 25441; 108 CFU). Primary outcomes were duration of diarrhea and the proportion diarrhea-free at 72 h. The secondary outcome measures included the proportion of diarrhea-free children during first 10th day of the study. A subgroup analysis for gut microbiota composition at Day 0, 10th and 30th days of the study have been performed.

**Results:**

Of 145 enrolled children, 79 in the probiotic group (34 girls, 45 boys) and 66 in the control (30 girls and 36 boys); baseline demographics were comparable. The duration of diarrhea was significantly reduced in the probiotic group compared to the control group (46.4 ± 29.6 h vs. 81.6 ± 38.5 h, *p* < 0.001). The percentage of diarrhea-free children was significantly larger in the probiotic group at 72 h compared to the control (86.0% vs. 33.3%, *p* < 0.001). Persistence of diarrhea was lower in the probiotic group at 24, 48, and 96 h (all *p* < 0.001) and at day 6 (2.5% vs. 15.1%; *p* < 0.05); by days 7–10, persistence was rare in both groups. The probiotic combination is well-tolerated, and no adverse events have been reported. Alpha diversity indices were unchanged within/between groups. Bray–Curtis and Jaccard PCoA showed no between-group separation; unweighted UniFrac revealed differences within the probiotic group (day 1 vs. day 30) and between groups at day 30 (*p* < 0.05). LEfSe indicated enrichment of taxa associated with recovery in the probiotic arm and control group, and there is difference between group at Day 30.

**Conclusion:**

This study evaluates a specific combination of *L. acidophilus* DSMZ 26280 and *L. reuteri* DSMZ 25441 in a randomized controlled setting, adding to the growing body of strain-specific probiotic research in pediatric acute infectious diarrhea. Adding probiotics to treatment is well-tolerated and reduces the duration of diarrhea by approximately 35 h when it starts in the early hours of infection. This probiotic combination use is associated with modest phylogenetics shifts in gut microbiota composition, with enrichment of certain taxa that have been previously associated with gut homeostasis in other contexts; however, their functional and clinical significance in this setting remains unclear. Larger blinded trials are warranted to confirm durability and detailed metagenomic analysis including metabolomics.

## 1 Introduction

Acute infectious diarrhea remains a significant cause of morbidity in pediatric populations (GBD 2021 Diarrhoeal Diseases Collaborators, 2025). Although generally self-limiting, acute infectious diarrhea can lead to dehydration, hospitalization, and, in severe cases, death, especially in younger children (Guarino et al., 2020, 2014). Probiotics, prebiotics, synbiotics, postbiotics, and combinations have been studied for their role in ameliorating the course of infectious diarrhea (Szajewska et al., 2023; Hojsak et al., 2023; Indrio et al., 2024; Szajewska and Salminen, 2023). Probiotics have been extensively studied for acute infectious diarrhea (Szajewska et al., 2023; Zemła et al., 2026). Potential clinical benefits of probiotics in pediatric acute infectious diarrhea are a reduction of diarrhea duration (by 1 or 1.5 days on average), especially effective when started early in the illness. In hospitalized children, effective probiotics have been associated with reduced hospital length of stay, approximately 1 day. Most probiotics used for acute infectious diarrhea in studies are well-tolerated with very few adverse effects (Szajewska et al., 2023; Zemła et al., 2026). Clinical guidelines from ESPGHAN (European Society for Pediatric Gastroenterology, Hepatology and Nutrition), and the World Gastroenterology Organization recommend the use of specific strains, including *Lacticaseibacillus rhamnosus* GG (LGG), *Saccharomyces boulardii* CNCM I-745, and *Limosilactobacillus reuteri* DSM 17938, for the management of acute gastroenteritis in children, emphasizing the importance of strain-specificity and quality control (Szajewska et al., 2023). Proposed potential mechanisms of action for probiotics include restoring intestinal microbiota disrupted by infection, strengthening gut epithelial barrier function, and attenuating intestinal inflammation (do Carmo M et al., 2018; Dinleyici et al., 2018; Poeta et al., 2024; Toro Monjaraz et al., 2021). However, microbiota analyses in children receiving probiotics for acute infectious diarrhea are limited, and these results are also probiotic-strain specific (Dinleyici et al., 2018; Toro Monjaraz et al., 2021).

While certain strains of *Lactobacillus acidophilus* and *L. reuteri* have demonstrated clinical benefits in acute diarrhea, no study has evaluated their combined use in acute infectious diarrhea. Since the effects of probiotic(s) are strain-specific, all combinations should be evaluated with randomized controlled trials (Szajewska et al., 2023; McFarland et al., 2018). We aim to evaluate the effects of a combination of *L. acidophilus* and *L. reuteri* on the clinical findings, and gut microbiota composition in children with acute infectious diarrhea.

## 2 Materials and methods

This prospective, randomized, controlled open-label clinical was conducted at the Pediatric Emergency Department of Prof. Dr. Ilhan Varank Sancaktepe Training and Research Hospital (Istanbul, Türkiye) between March 2024 and August 2024. The study protocol was reviewed and approved by the Ethics Committee of Prof. Dr. Ilhan Varank Sancaktepe Training and Research Hospital (approval date: April 17, 2024; decision no: 126). Written informed consent was obtained from the legal guardians of all participating children prior to enrollment. The study was conducted in accordance with the principles of the Declaration of Helsinki.

Inclusion criteria were children aged 1–6 years, clinical diagnosis of acute gastroenteritis, presenting within 24 h of symptom onset, no or mild dehydration, and availability of written informed consent from parents/guardians Children with antibiotic use within the preceding month and those with malnutrition were excluded to minimize potential confounding effects on clinical outcomes and gut microbiota composition. Children who have moderate or severe dehydration, immunosuppression or chronic systemic disease were also excluded.

Participants were randomly assigned in a 1:1 ratio to either the intervention or control group using a computer-generated randomization program with variable block sizes of 8. This was an open-label study; investigators and parents are not blinded. Intervention group received standard therapy (oral rehydration solution ± intravenous fluids), plus a probiotic preparation containing *L. acidophilus* DSMZ 26280; 108 CFU and *L. reuteri* DSMZ 25441; 108 CFU, administered orally as five drops daily for 5 consecutive days. Control group received standard therapy consisting of oral rehydration solution and/or intravenous fluid. All participants received standardized dietary and supportive care recommendations. Caregivers were advised to continue age-appropriate feeding, in addition to oral rehydration therapy. The use of additional supplements, including other probiotic, prebiotic, synbiotic, or postbiotic products, was not permitted during the study period.

The primary outcomes were the duration of diarrhea (in days) and the proportion of diarrhea free children at the 72 h of intervention. Secondary endpoints percentage of children with diarrhea during first 10 days. Participants were closely monitored throughout the intervention period. Any adverse events were documented, and appropriate clinical management was provided. In a randomly selected subgroup of children, gut microbiota analysis has been performed before and after intervention.

For microbiota analysis, stool samples were collected at day 0, day 10, and day 30. Microbial DNA was extracted using the DiaRex^®^ Stool Genomic DNA Extraction Kit. DNA concentrations were measured with Qubit fluorometry and Nanodrop spectrophotometry. The 16S rRNA gene was amplified and sequenced on the Illumina MiSeq platform. Data processing and analysis were performed with QIIME2 and R software, including assessment of alpha and beta diversity and LEfSe analysis.

Statistical Analysis: Sample size (*n* = 160; 80 per group) was calculated to detect clinically meaningful differences with 80% power and 5% types I error rate. All statistical analyses were performed using JASP statistical software (JASP Team, 2025, JASP Version 0.95.3). Descriptive statistics were presented as mean, median, minimum, and maximum values, as appropriate. Categorical variables were compared with Pearson's chi-square test, and correlations between quantitative variables were assessed using Spearman's correlation coefficient. A *p*-value < 0.05 was considered statistically significant.

## 3 Results

A total of 145 children were enrolled in the study, with 79 (45 boys and 34 girls) in the probiotic group and 66 (30 girls and 36 boys) in the control group. Baseline demographic and clinical characteristics were comparable between groups (Table 1). The mean age was 57.1 ± 35.3 months in the probiotic group and 66.0 ± 38.9 months in the control group, with no significant difference observed (*p* > 0.05). The median number of stools during the 24 h prior to enrollment was similar between the study groups (*p* > 0.05).

**Table 1:** Demographic and clinical findings of the probiotic group and control group.

Demographic and clinical characteristics	Probiotic group (*n* = 79)	Control group (*n* = 66)	*p*
Gender (Girls/Boys)	34 girls, 45 boys	30 girls,36 boys	*p* > 0.05
Age (months)^*^	57.1 ± 35.3	66.0 ± 38.9	*p* > 0.05
Median number of stools during the 24 h prior to inclusion^*Y*^	9 (5–20)	9 (5–12)	*p* > 0.05
Duration of diarrhea (hours)^*^	46.4 ± 29.6 h	81.6 ± 38.5 h	***p*** **<** **0.001**

Bold values: statistically significant.

^*^Values are presented as mean ± standard deviation (SD).

^Y^Values are presented as median (minimum–maximum).

### 3.1 Clinical findings

The duration of diarrhea was significantly shorter in the probiotic group compared with the control group (46.4 ± 29.6 h vs. 81.6 ± 38.5 h, respectively; *p* < 0.001; Table 1). The proportion of children with ongoing diarrhea significantly differed between groups during the early study period (Table 2). At the 24 h, persistent diarrhea was observed in 58.2% of the probiotic group compared to 93.9% of the control group (RR 0.61; 95% CI: 0.50–0.75; *p* < 0.001). By the 48th hour, diarrhea persisted in 15.2% of the probiotic group vs. 77.2% in the control group (RR 0.19; 95% CI: 0.11–0.33; *p* < 0.001). At the 72 h, only 13.9% of children in the probiotic group had diarrhea compared with 66.7% in the control group (RR 0.20; 95% CI: 0.11–0.37; *p* < 0.001). Similarly, at 96 h, persistence was observed in 11.4% vs. 51.5% of children, respectively (RR 0.22; 95% CI: 0.11–0.42; *p* < 0.001). These findings demonstrate a rapid and clinically meaningful separation between groups, with the probiotic intervention significantly reducing the proportion of children with ongoing diarrhea as early as 24 h and maintaining this effect through 96 h. At the 120th hour, diarrhea remained in 6.3% of the probiotic group and 16.7% of the control group (RR 0.37; 95% CI: 0.13–1.03), with a trend toward significance (*p* = 0.059). By the 6th day, the difference became significant again (2.5% vs. 15.1%; RR 0.16; 95% CI: 0.03–0.73; *p* < 0.05). At later time points (days 7–10), diarrhea was rare in both groups, and no statistically significant differences were detected (all *p* > 0.05; Table 2). We did not observe adverse events related with the probiotic group. The number needed to treat to achieve one additional child free from diarrhea was two at 48 and 72 h and three at 96 h, demonstrating a strong and clinically meaningful treatment effect.

**Table 2:** Proportion of children with ongoing diarrhea at predefined time points in the probiotic and control groups.

Assessment time point	Probiotic group (*n* = 78)	Control group (*n* = 66)	RR (95% CI)	*p*
24 h	46/79 (58.2%)	62/66 (93.9%)	0.61 (0.50–0.75)	***p*** **<** **0.001**
48 h	12/79 (15.2 %)	51/66 (77.2%)	0.19 (0.11–0.33)	***p*** **<** **0.001**
72 h	11/79 (13.9%)	44/66 (66.7%)	0.20 (0.11–0.37)	***p*** **<** **0.001**
96 h	9/79 (11.4%)	34/66 (51.5%)	0.22 (0.11–0.42)	***p*** **<** **0.001**
120 h	5/79 (6.3%)	11/66 (16.7%)	0.37 (0.13–1.03)	*p* = 0.059
6th day	2/79 (2.5%)	10/66 (15.1%)	0.16 (0.03–0.73)	***p*** **<** **0.05**
7th day	1/79 (1.27 %)	4/66 (6.0%)	0.20 (0.02–1.82)	*p* = 0.15
8th day	0/79	2/66 (3.0%)	0.16 (0.008–3.42)	*p* = 0.24
9th day	0/79	2/66 (3.0%)	0.16 (0.008–3.42)	*p* = 0.24
10th day	0/79	2/66 (3.0%)	0.16 (0.008–3.42)	*p* = 0.24

Bold values: statistically significant.

Data are presented as number of children with diarrhea over total number of children in each group, with corresponding percentages.

Relative risks (RR) with 95% confidence intervals (CI) were calculated comparing the probiotic group to the control group at each time point.

### 3.2 Gut microbiota analysis

Metagenomic analysis of stool samples was performed in 10 patients from the probiotic group and 10 patients from the control group at day 0, day 10, and day 30.

**Figure 1:**
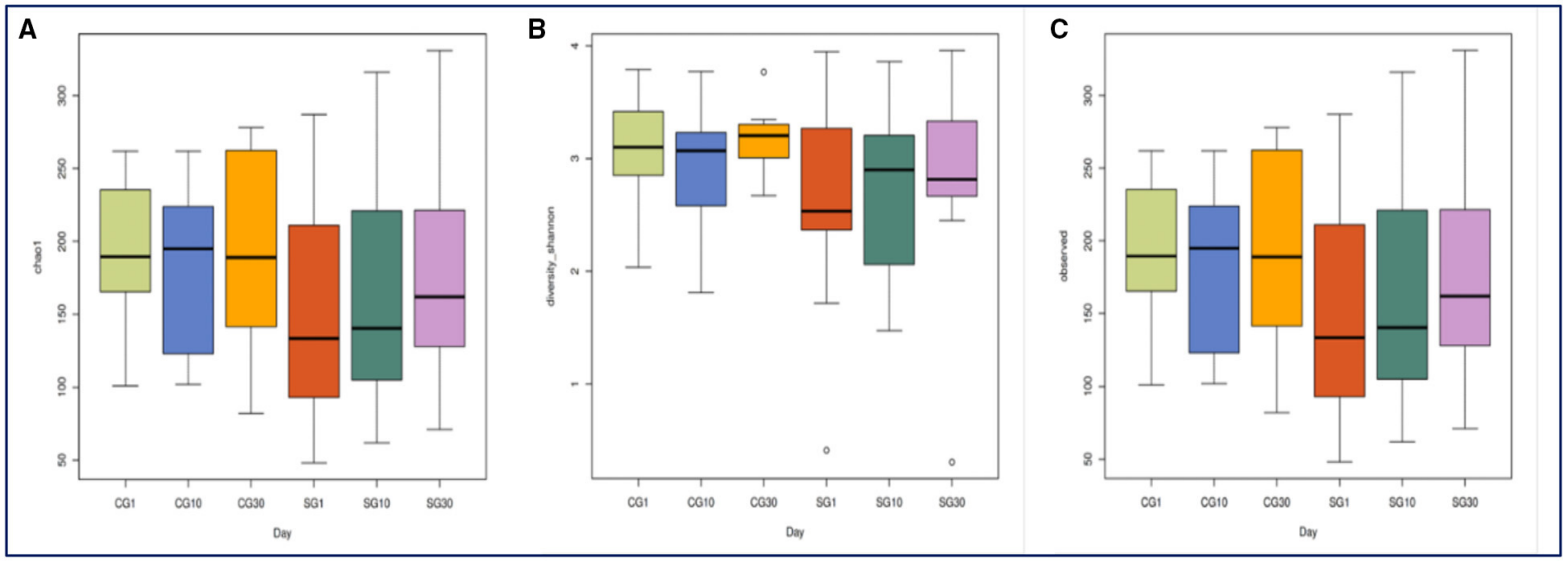
Alpha diversity indices of the intestinal microbiota in the study and control groups. Boxplots represent alpha diversity indices of stool samples collected from the study group (SG) and control group (CG) at day 0, day 10, and day 30. **(A)** Chao1 richness estimator, reflecting the estimated number of taxa within samples. **(B)** Shannon diversity index, combining both richness and evenness of microbial communities. **(C)** Observed ASV counts, indicating the number of distinct operational taxonomic units detected. Across all three indices, no significant differences were observed either within groups over time or between the probiotic (SG) and control (CG) groups (*p* > 0.05 for all comparisons).

**Figure 2:**
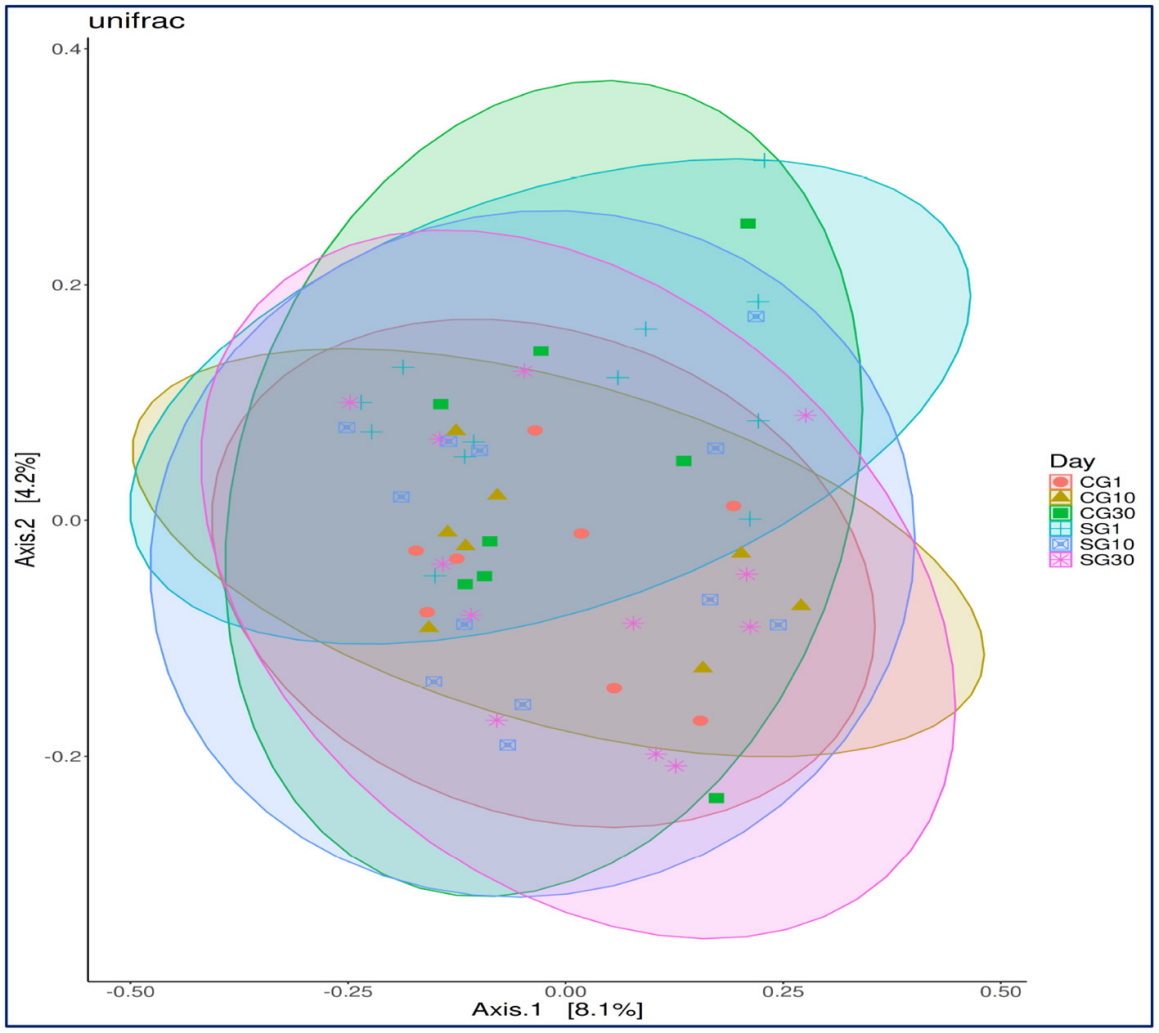
Beta diversity of gut microbiota based on unweighted UniFrac distances. Principal coordinate analysis (PCoA) plots display the microbial community structure in stool samples collected from the study group (SG) and control group (CG) at day 0, day 10, and day 30. Each point represents the microbial composition of an individual sample, while colored ellipses indicate 95% confidence intervals for each group and timepoint. Unweighted UniFrac analysis demonstrated significant differences within the probiotic group between day 0 and day 30, and between the study and control groups at day 30 (*p* < 0.05).

Alpha diversity, which reflects the microbial diversity within individual samples, was assessed using observed ASV counts, the Chao1 index, and the Shannon index. Across all indices, no significant differences were observed either within groups over time or between the probiotic and control groups (*p* > 0.05 for all comparisons; Figure 1). Beta diversity analyses were performed using Bray–Curtis and Jaccard distance–based principal coordinate analysis (PCoA). No significant differences were observed between the probiotic and control groups at day 0, day 10, or day 30. However, unweighted UniFrac PCoA revealed significant differences within the probiotic group between day 1 and day 30, as well as between the probiotic and control groups at day 30 (*p* < 0.05 for both comparisons; Figure 2).

**Figure 3:**
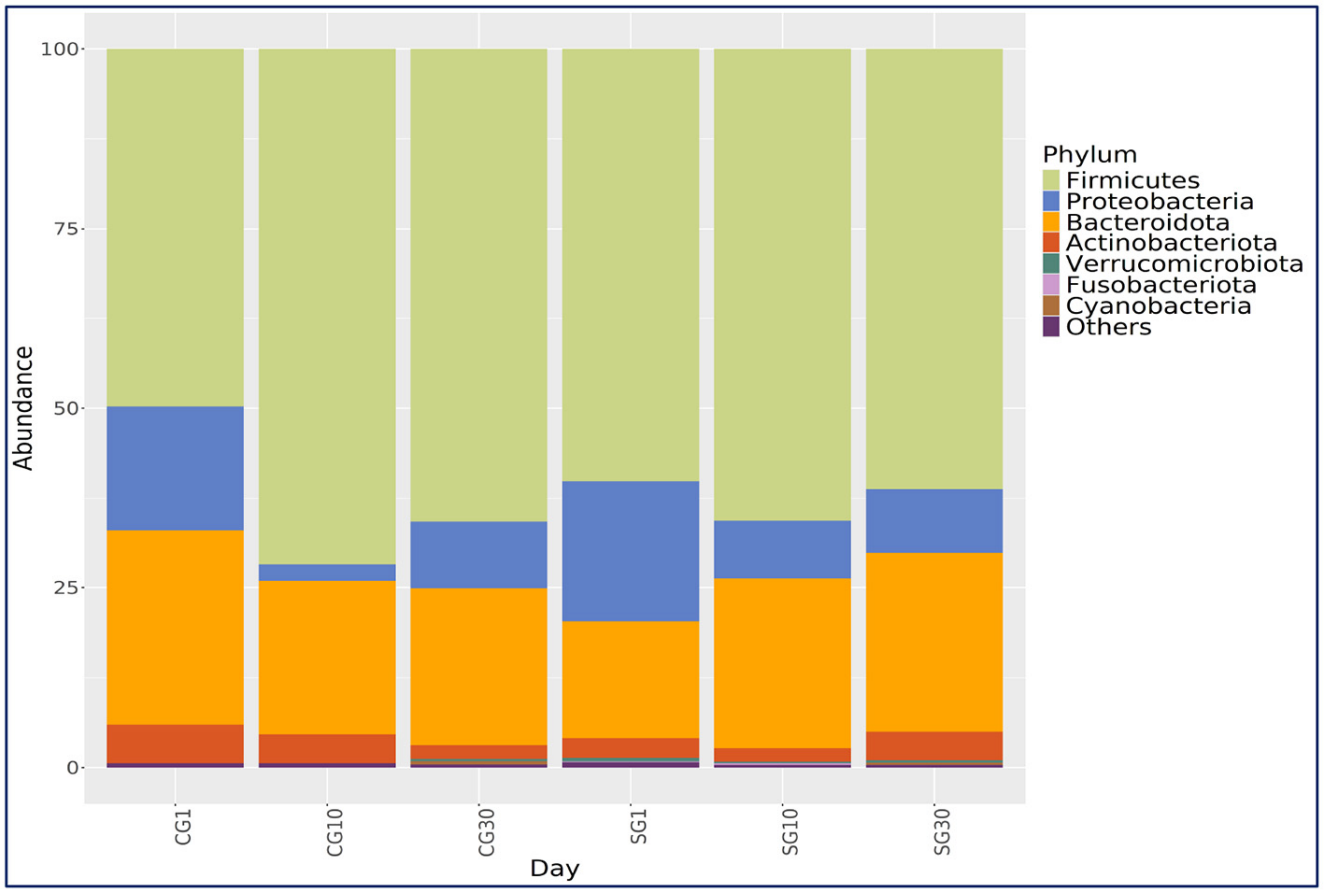
Phylum-level composition of the gut microbiota in the probiotic and control groups over time. Stacked bar plots illustrate the relative abundance (%) of the major bacterial phyla in stool samples collected from the control group (CG) and the probiotic group (SG) at day 0, day 10, and day 30. Bars represent the proportional contribution of each phylum to the total microbial community within each group and time point.

At the phylum level, Firmicutes were dominant in both the probiotic and control groups. At day 0, the probiotic group showed Firmicutes (62.4%), Bacteroidetes (16.5%), Proteobacteria (17.3%), and Actinobacteria (2.7%), while the control group had Firmicutes (49.1%), Bacteroidetes (27.7%), Proteobacteria (17.2%), and Actinobacteria (5.6%; Figure 3).

**Figure 4:**
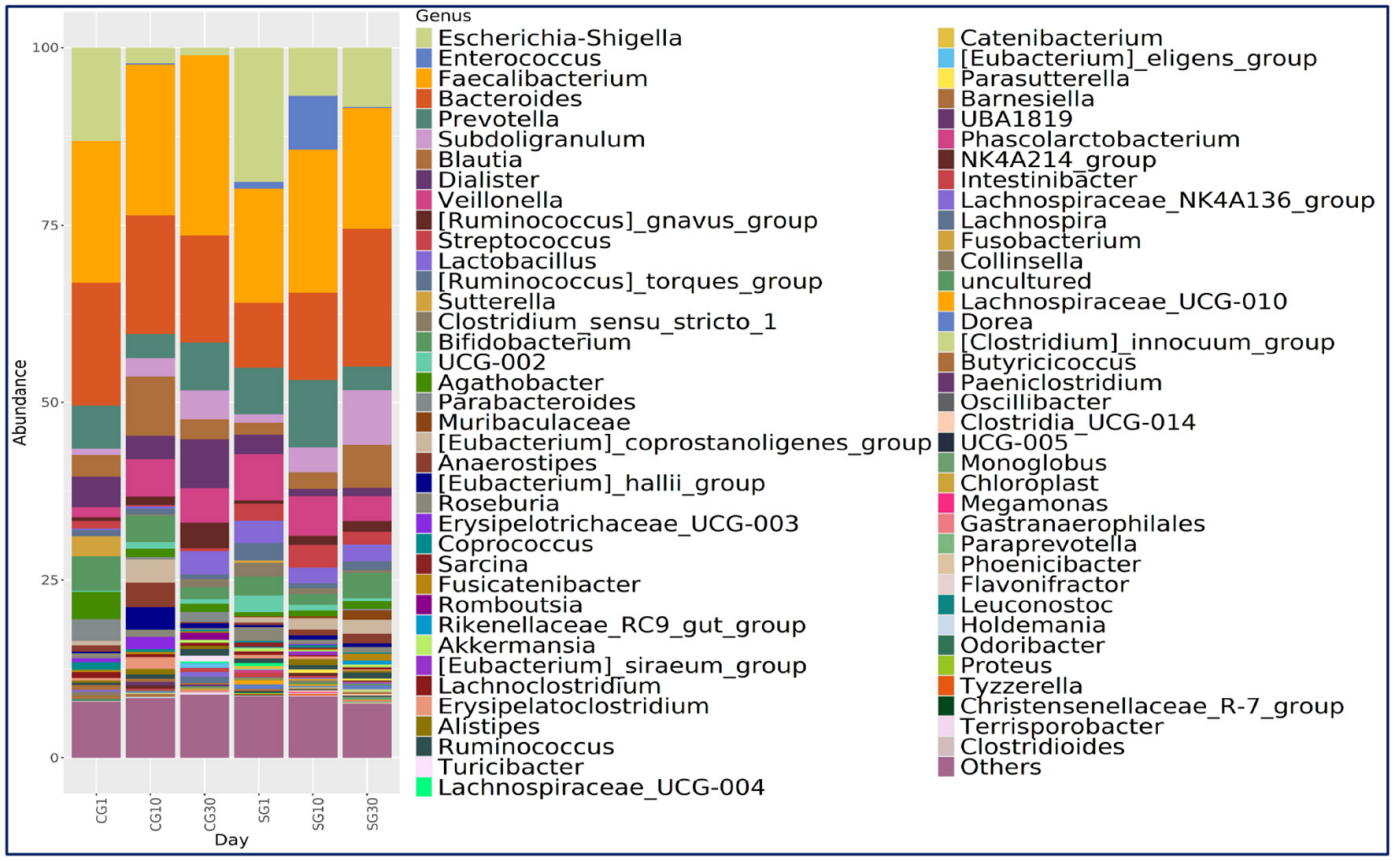
Genus-level composition of the gut microbiota in the probiotic and control groups over time. Stacked bar plots illustrate the relative abundance (%) of the major bacterial genera in stool samples collected from the control group (CG) and the probiotic group (SG) at day 0, day 10, and day 30. Bars represent the proportional contribution of each genera to the total microbial community within each group and time point.

At the genus level, the probiotic group at day 0 was characterized by *Faecalibacterium* (18.1%), *Escherichia–Shigella* (16.8%), *Bacteroides* (8.8%), *Prevotella* (7.2%), *Veillonella* (5.9%), *Dialister* (3.1%), and *Lactobacillus* (3.0%). In the control group showed *Faecalibacterium* (19.8%), *Bacteroides* (17.6%), *Escherichia–Shigella* (12.7%), *Prevotella* (6.6%), *Bifidobacterium* (5.0%), and *Dialister* (4.5%; Figure 4).

**Figure 5:**
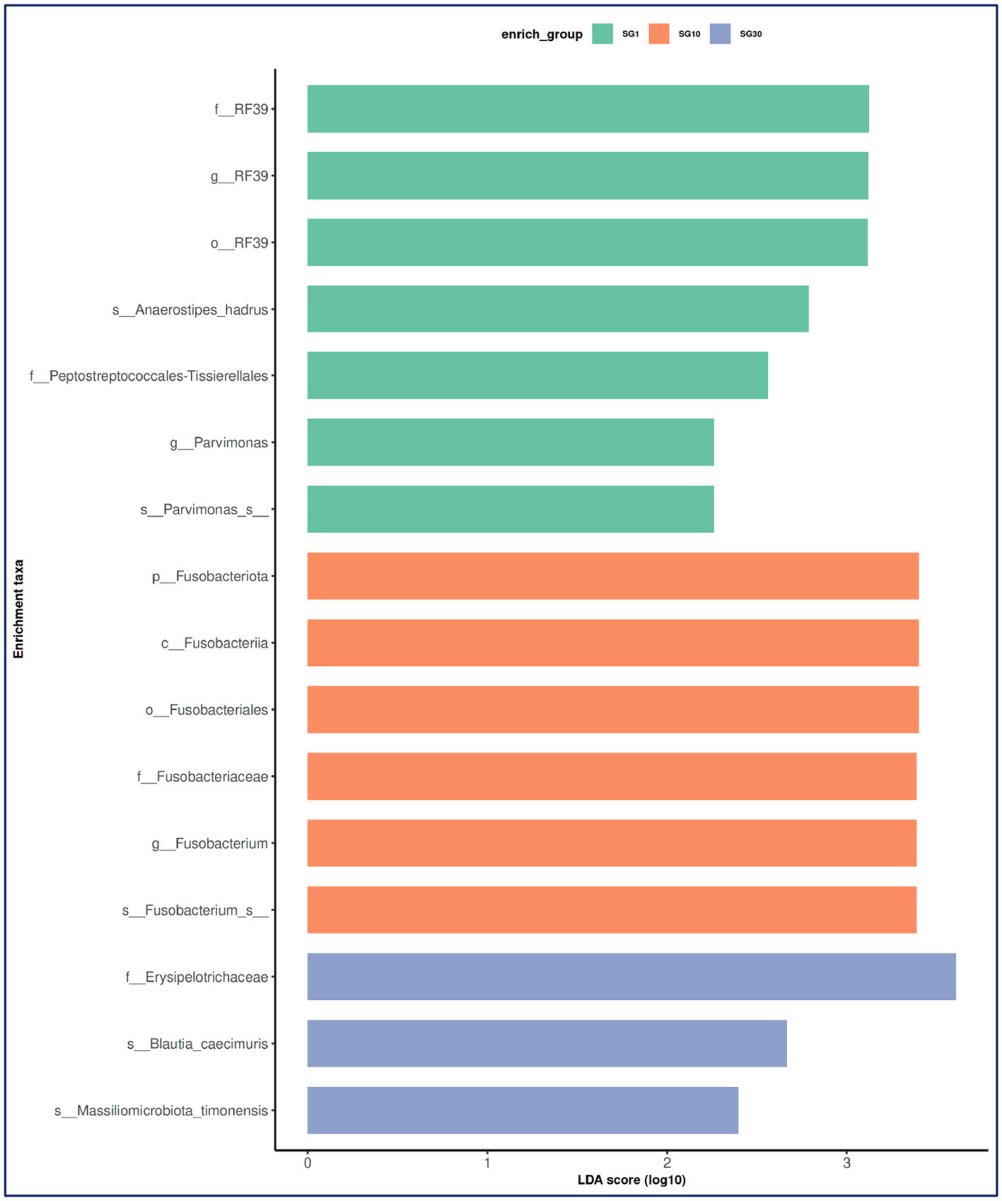
LEfSe analysis showing longitudinally enriched taxa in the probiotic group. Linear discriminant analysis effect size (LEfSe) identifies bacterial taxa that were differentially abundant within the probiotic group at day 0 (SG1), day 10 (SG10), and day 30 (SG30). Bars represent the logarithmic LDA score (log10), reflecting the effect size of each taxon. Taxa enriched at day 0 (SG1; green); at day 10 (SG10; orange), by day 30 (SG30; blue). Only taxa with LDA scores >2 and *p* < 0.05 are shown.

To determine significant bacterial taxa differentiating groups, linear discriminant analysis effect size (LEfSe) was applied (LDA score > 2, *p* < 0.05). At day 0, no significant differences were found between the probiotic and control groups. Within the probiotic group, LEfSe analysis revealed that *Parvimonas* was enriched at day 0 (LDA score = 3.297, *p* = 0.017). By day 10, *Fusobacteriota* at the phylum level (LDA score = 2.259, *p* = 0.040) and *Fusobacterium* at the genus level (LDA score = 3.388, *p* = 0.029) were enriched. By day 30, *Blautia caecimuris* (LDA score = 2.665, *p* = 0.041) and *Massilomicrobiota timonensis* (LDA score = 2.395, *p* = 0.033) were identified as dominant taxa. In the control group, LEfSe analysis indicated enrichment of *Proteobacteria* (LDA score = 4.960, *p* = 0.003) and *Escherichia–Shigella* (LDA score = 4.880, *p* = 0.043) at day 0. By day 30, *Firmicutes* (LDA score = 5.270, *p* = 0.047), *Rothia* at the genus level (LDA score = 2.532, *p* = 0.001), and *Rothia mucilaginosa* at the species level (LDA score = 2.506, *p* = 0.037) were significantly enriched (Figure 5).

**Figure 6:**
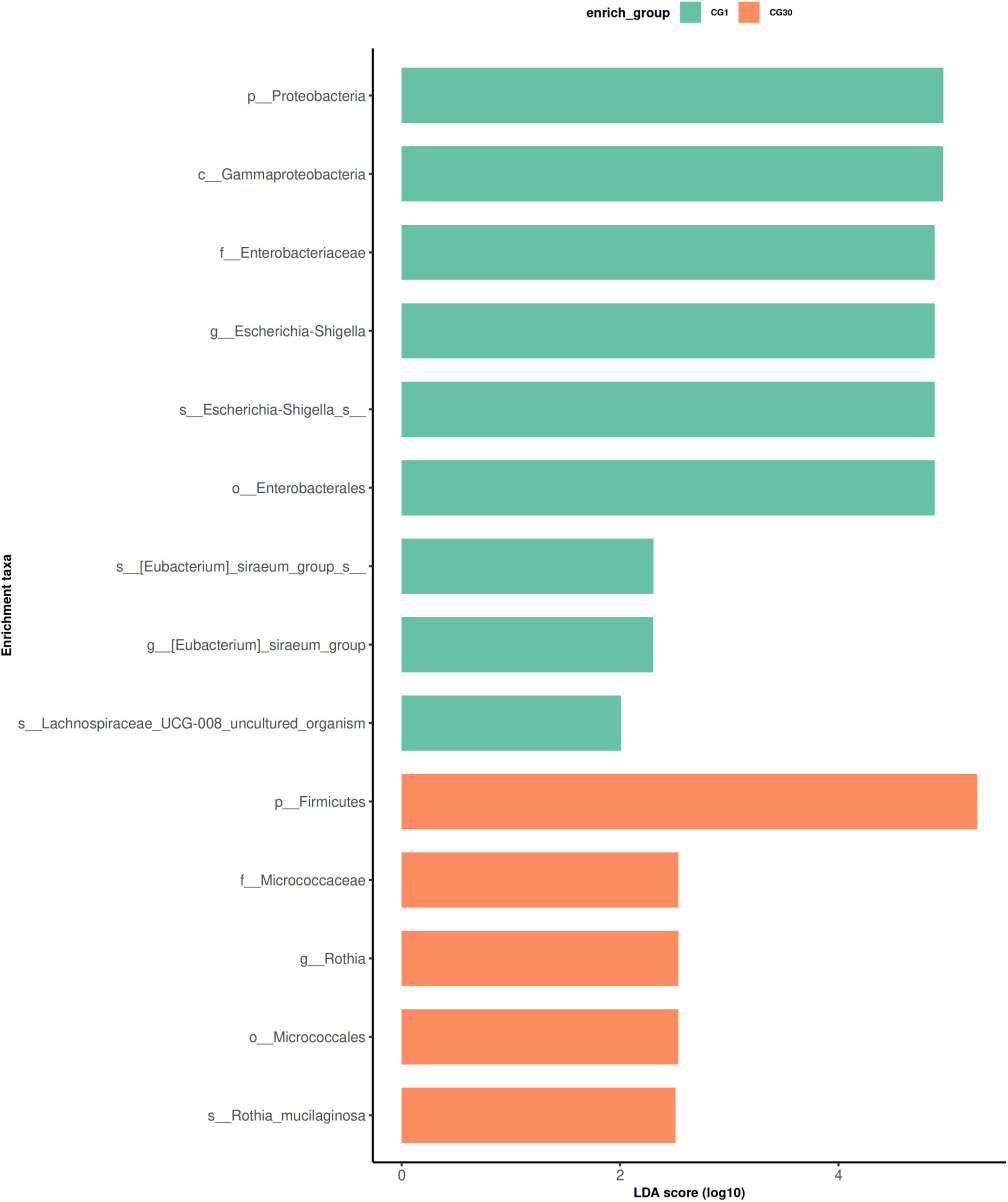
LEfSe analysis showing longitudinally enriched taxa in the control group. Linear discriminant analysis effect size (LEfSe) identifies bacterial taxa that were differentially abundant within the control group at day 0 (CG1) and day 30 (CG30). Bars represent the logarithmic LDA score (log10), indicating the magnitude of each taxon's contribution to group separation. At day 0 (CG1; green), by day 30 (CG30; orange). Only taxa with LDA scores >2 and *p* < 0.05 are shown.

When comparing the probiotic and control groups at day 30, *Akkermansia* sp. (LDA score = 3.542, *p* = 0.025), *Alistipes finegoldii* (LDA score = 3.004, *p* = 0.025), and *Sutterella* spp. (LDA score = 2.148, *p* = 0.046) were more abundant in the probiotic group. In contrast, *Selimonas* spp. (LDA score = 2.607, *p* = 0.042) and *Eubacterium siraeum* group spp. (LDA score = 2.477, *p* = 0.042) were enriched in the control group (Figure 6).

## 4 Discussion

In this study, a once daily single dose *L. acidophilus* and *L. reuteri* combination treatment for 5 days significantly shortened the duration of diarrhea in children with acute infectious diarrhea. The clinical benefit emerged early by 24 h and by 48 h diarrhea persist in only 15.2%, indicating rapid symptom improvement. At the 72 h, only 13.9% of children in the probiotic group still had diarrhea compared with 66.7% in the control group, and the between-group separation persisted through 96 h. The combination was well-tolerated, and no treatment related-adverse events were observed. These results align with our consistent evidence from randomized trials conducted in Türkiye over the past decade showing that strain-specific probiotics or synbiotics, when added to standard oral rehydration therapy, accelerate recovery in pediatric acute infectious diarrhea with a characteristic onset of benefit within the first 24–48 h and an excellent safety profile (Dinleyici et al., 2013; Dinleyici and PROBAGE Study Group, 2014; Dinleyici et al., 2015a,b). In outpatient settings, *L. reuteri DSM 17938* reduced diarrhea duration by approximately 15 h and decreased the proportion of children with diarrhea at 48 h, although group differences diminished after 72 h (Dinleyici et al., 2015a). In hospitalized children, the same strain yielded a more pronounced effect, with significantly more diarrhea-free children by 24–72 h and a reduction in length of hospital stay exceeding 1 day (Dinleyici and PROBAGE Study Group, 2014). Similarly, in our large multicenter trial of *S. boulardii* CNCM I-745 (*n* = 363), diarrhea duration was shortened by approximately 24 h across outpatient, emergency care, and inpatient settings, with clear separation between groups emerging at 48–72 h and substantial reductions in both emergency care and hospital length of stay (Dinleyici et al., 2015b). Our multispecies synbiotic trial (six probiotic strains plus fructooligosaccharides) showed one of the largest clinical effects, reducing diarrhea duration by approximately 36 h and shortening hospitalization by nearly 1 day, with benefits evident from 24 h onward (Dinleyici et al., 2013). Against this background, the current once-daily *L. acidophilus* + *L. reuteri* combination produced a comparably large reduction in diarrhea duration (~35 h) and an early, sustained separation between groups, from 24 to 96 h, supporting the concept that early initiation and complementary strain pairing may maximize clinical impact. The magnitude of effect observed in this study, particularly within the first 48–72 h, is clinically relevant, as early symptom resolution may reduce dehydration risk, caregiver burden, and healthcare utilization. In a 2022, systematic review and meta-analysis pooling 15 randomized controlled trials (*n* = 1,765) adding *L. acidophilus* to standard rehydration was associated with a shorter diarrhea duration vs. placebo/no probiotic, but the benefit was less consistent when *L. acidophilus* was given as a single strain, suggesting that efficacy is more consistent when *L. acidophilus* is used in combination products (Cheng et al., 2022). Probiotic therapy in pediatric acute infectious diarrhea has been extensively studied, with evidence supporting strain-specific effects on clinical outcomes. The present study adds to this body of evidence by evaluating a specific combination of *L. acidophilus* DSMZ 26280 and *L. reuteri* DSMZ 25441, which has not been previously studied in this clinical context. Therefore, our results should be considered as incremental but clinically relevant, particularly in the context of strain-specific probiotic research.

Microbiome analyses in pediatric acute diarrhea remain limited, and most available data suggest that clinically meaningful recovery can occur with either global diversity restoration or more selective ecological shifts. In the present study, the lack of significant differences in alpha diversity within or between groups suggests that overall richness and evenness were broadly preserved over follow-up despite clear clinical improvement. Similarly, the absence of separation by Bray–Curtis and Jaccard PCoA indicates that large abundance-weighted shifts in dominant taxa were not the primary signature of treatment. In contrast, the unweighted UniFrac signal—showing change within the probiotic group over time and separation from controls at day 30—suggests phylogenetic restructuring driven by presence/absence changes, potentially involving low-abundance lineages. Because unweighted UniFrac is sensitive to lineage turnover rather than relative abundance, these findings support a model in which probiotics “nudge” the ecosystem through selective gain/loss of specific taxa, rather than producing wholesale changes in community-wide diversity. This selective restructuring is biologically plausible during post-infectious recovery and may represent restoration of resilience (e.g., reduction of aerotolerant pathobionts and re-emergence of health-associated anaerobes), even when summary diversity metrics remain stable.

Our microbiome observations can be interpreted alongside prior longitudinal studies. In the FACID time-series study of rotavirus diarrhea treated with *S. boulardii* CNCM I-745, Dinleyici et al. (2018) children exhibited reduced alpha diversity and compositional separation from healthy peers during the acute phase, followed by convergence toward a healthy-like state by days 10–30, accompanied by increased temporal instability early in illness and enrichment of *Proteobacteria* (particularly *Gammaproteobacteria*) during diarrhea (Dinleyici et al., 2018). Similarly, the Mexico City observational study reported reduced alpha diversity and marked beta-diversity separation during acute diarrhea at presentation, with recovery toward a control-like microbiota by day 15; exploratory analyses suggested that *S. boulardii* CNCM I-745 use may facilitate diversity restoration, although non-random treatment allocation and small comparator groups limited inference (Toro Monjaraz et al., 2021). Together, these studies reinforce that acute infectious diarrhea is characterized by a transient dysbiosis and ecological instability that improves over time, while the specific microbiome signature and its recovery trajectory may vary with pathogen spectrum, host factors, and treatment timing. In our dataset, LEfSe-based signals indicated time-dependent differences in selected taxa, and between-group distinctions at day 30 (e.g., enrichment of taxa such as *Akkermansi*a, *Alistipes*, and *Sutterella* in the probiotic group) may point to potential mucosal or immunomodulatory associations; however, mechanistic interpretation remains cautious given the limited functional resolution of 16S profiling and the incomplete biological characterization of several taxa. In contrast, the control group was characterized by enrichment of *Sellimonas* spp. and *Eubacterium siraeum* group spp. Both *E. siraeum* and *Sellimona*s *intestinalis* are anaerobic, *Sellimonas* is more often discussed as a recovery / biomarker taxon rather than a driver of phenotype. In 2020, Muñoz et al. (2020) published comprehensive genome analyses of *Sellimonas intestinalis*, a potential biomarker of homeostasis gut recovery. Although little is known about this *Lachnospiraceae* family member, its increased abundance has been reported in patients who have recovered from intestinal homeostasis after dysbiosis events. These findings provide the basis of knowledge about the potential of *S. intestinalis* as a bioindicator of intestinal homeostasis recovery and contribute to advancing the characterization of gut microbiota members with beneficial potential. Taken together, these findings suggest that probiotic intervention did not markedly alter overall diversity metrics but influenced specific microbial lineages in a manner that may support more favorable recovery dynamics following acute infectious diarrhea. The microbiota findings of this study should be interpreted cautiously. Given the small sample size and the use of 16S rRNA sequencing, these analyses were exploratory and not powered to detect subtle or strain-level differences. No functional analyses or correlation between microbial composition and clinical outcomes were performed; therefore, the clinical relevance of specific taxa identified in LEfSe analysis should be interpreted with caution.

This study has some limitations. First, it was conducted at a single center, which may limit the generalizability of the findings across different geographic regions, healthcare settings, and pathogen distributions. The epidemiology and clinical course of acute infectious diarrhea may vary depending on regional pathogen prevalence, socioeconomic factors, and healthcare practices. Although the study was performed in a high-volume tertiary pediatric emergency department with a heterogeneous patient population and standardized management protocols, these findings should be interpreted with caution. Multicenter studies involving diverse populations and pathogen spectra are needed to confirm the external validity of these results. The open-label design may introduce performance and reporting bias, particularly for symptom-based endpoints. Although the study was open-label, the primary outcome (time to last diarrheal stool) is objective and unlikely to be influenced by reporting bias. Another limitation of this study is the lack of systematic pathogen identification. As the clinical course of acute infectious diarrhea may vary depending on the underlying etiology (e.g., viral vs. bacterial pathogens), the absence of microbiological confirmation precluded pathogen-specific subgroup analyses. However, randomization and early enrollment (< 24 h from symptom onset) likely minimized imbalance between groups. Future studies incorporating multiplex molecular diagnostics are warranted to better elucidate pathogen-specific responses to probiotic interventions. The microbiota part of this study included a limited number of participants, which restricts statistical power and increases the risk of type I and type II errors. Furthermore, 16S rRNA sequencing does not allow strain-level tracking; therefore, it is not possible to directly link the administered probiotic strains to observed microbial changes. This exploratory longitudinal microbiome part was designed to identify phylogenetic signatures of recovery rather than detect small abundance differences. Finally, 16S rRNA sequencing provides limited functional inference; metagenomic and metabolomic profiling, alongside longer clinical follow-up, would better clarify whether observed phylogenetic restructuring translates into durable functional recovery or longer-term outcomes.

## 5 Conclusions

In conclusion, once daily 5-day course of *L. acidophilus* DSMZ 26280 and *L. reuteri* DSMZ 25441 accelerated early clinical recovery in pediatric acute infectious diarrhea, with excellent tolerability and microbiome signals consistent with selective phylogenetic restructuring rather than broad diversity expansion. Given the absence of strain-level resolution and functional profiling, these taxonomic shifts should be considered descriptive rather than mechanistic. These findings provide a rationale for future multicenter, geographically diverse randomized controlled trials evaluating strain-specific probiotic combinations in pediatric acute infectious diarrhea. Larger multicenter, placebo-controlled trials with standardized endpoints and integrated metagenomic/metabolomic analyses are warranted to confirm effect size, durability, and mechanistic pathways.

## Data Availability

The datasets generated and/or analyzed during the current study are publicly available in the NCBI repository (https://www.ncbi.nlm.nih.gov/bioproject) under BioProject accession number PRJNA1469248.
